# Localization of TGF-β type II receptor and ED-A fibronectin in normal conjunctiva and failed filtering blebs

**Published:** 2008-01-25

**Authors:** Tobias Meyer-ter-Vehn, Franz Grehn, Günther Schlunck

**Affiliations:** Department of Ophthalmology, University of Würzburg, D 97080 Würzburg, Germany

## Abstract

**Purpose:**

The cytokine transforming growth factor-β (TGF-β), and the ED-A splice variant of the extracellular matrix protein fibronectin modulate wound healing and scar formation. To further elucidate their possible role in filtering bleb scarring after glaucoma surgery in human eyes in vivo, we studied the cell type specific localization of TGF-β receptors and the presence of ED-A fibronectin in sections of normal conjunctiva and scarred filtering blebs.

**Methods:**

Cryosections of normal conjunctiva (four patients) and scarred filtering blebs (seven patients) were studied by double-label immunofluorescence. Antibodies against PECAM-1 and prolyl-4-hydroxylase allowed for specification of vascular endothelial cells and activated fibroblasts, respectively. TGF-β receptor type II (TGF-β-RII), α-smooth muscle actin, O-linked sialoglycoprotein, fibronectin and the ED-A fibronectin splice-variant were also detected using specific antibodies. Labeled sections were viewed with a confocal laser scanning microscope.

**Results:**

Vascular endothelial cells expressed TGF-β-RII in both normal and scarred tissue. TGF-β-RII was sparsely detected in the fibroblasts of normal conjunctiva while it was strongly expressed in most fibroblasts of the scarred filtering blebs. Similarly, ED-A fibronectin was not detected in the extracellular matrix of normal conjunctiva but abundantly present in scarred filtering blebs.

**Conclusions:**

Filtering bleb scarring is associated with an abundant expression of TGF-β receptors in activated fibroblasts and the deposition of the fibrogenic ED-A fibronectin splice-variant. These data support the concept of targeting TGF-β signaling to prevent scar formation after filtering glaucoma surgery.

## Introduction

Scar formation is the most frequent cause of failure following glaucoma filtering surgery, but the pathophysiological mechanisms of filtering bleb scarring are not fully elucidated. The cytokine transforming growth factor-β (TGF-β), is pivotal in wound healing and scar formation in general [1,2]. All three TGF-β isoforms have been identified in the eye [3,4] with TGF-β-2 being the predominant isoform associated with ocular scarring diseases such as proliferative vitreoretinopathy and cataract formation [5,6]. In a mouse model of conjunctival scarring, TGF-β-2 was strongly expressed in the stroma of the wounded area [7] and a TGF-β-2-specific neutralizing antibody prevented conjunctival scarring after glaucoma surgery in a rabbit model [8]. However, a recent phase III clinical trial failed to demonstrate a significant effect of TGF-β-2-specific antibodies in the prevention of filtering bleb scarring [9] and raised some reservations concerning the targeting of TGF-β.

In the early phases of wound healing, TGF-β is secreted by inflammatory cells [10] and acts as a chemoattractant. In the long-term, TGF-β promotes the transdifferentiation of fibroblasts to highly contractile myofibroblasts, which deposit extracellular matrix proteins and serve as the main agents of scarring if present persistently. TGF-β binds to a heterodimeric receptor complex, consisting of two serine-threonine kinase receptors designated TGF-β type I and II receptor, and leads to the activation of several intracellular signaling pathways. The tissue distribution of TGF-β receptor-bearing cells in normal conjunctiva and scarred filtering blebs is currently unclear.

Furthermore, scarring is associated with alterations in the extracellular matrix composition. TGF-β induces the expression of the ED-A fibronectin splice variant [11] as a precondition for TGF-β-mediated myofibroblast transdifferentiation. Studies in vitro revealed that TGF-β-induced myofibroblast transdifferentiation was averted by the blocking of ED-A fibronectin with specific antibodies [11]. The role of ED-A fibronectin in filtering bleb scarring has not been addressed.

To gain further insight into the possible role of TGF-β in filtering bleb scarring in human eyes in vivo, we studied the cell type-specific distribution of TGF-β-RII and the presence of ED-A fibronectin in normal conjunctiva and scarred filtering blebs.

## Methods

### Tissue samples

Conjunctival specimens were obtained during standard intraocular surgery after comprehensive information and written consent of the selected patients. The tenets of the Declaration of Helsinki were followed, and an institutional ethics committee approval had been granted. Native conjunctival tissue was gained from four patients undergoing strabismus surgery while hypertrophic scar tissue was obtained from seven patients undergoing filtering bleb revision surgery after multiple preceding operations (Table 1). All specimens were embedded in Tissue Tek cryopreservant and snap frozen in liquid nitrogen.

**Table 1:** Patient characteristics

Age [yrs]	Sex	Diagnosis	Previous Surgery [#of proc.]	Time after previous surgery [mo]	History of topical medication
14	f	Strabismus	0	-	-
29	f	Strabismus	0	-	-
6	m	Strabismus	0	-	-
28	m	Strabismus	0	-	-
53	m	Glaucoma / POAG	4	2	+ / multiple Allergies
8	m	2° Glaucoma after injury	5	1	+ Hx of 6 years
62	m	2° Glaucoma after injury	5	1	+ / Hx of 16yrs
79	f	Glaucoma / PEX	1	2	+ / Hx of 3yrs
37	f	Glaucoma / juvenile	4	8	+ / Hx of 14yrs
53	m	Glaucoma / uveitic	2	2	+ / Hx of 5yrs

Survey of patient characteristics including age, sex, reason for surgery (strabismus surgery or glaucoma surgery), type of glaucoma, number and time after previous surgery, history of topical medication. All patients undergoing revision glaucoma surgery received topical mitomycin C treatment. Abbreviations: POAG: primary open angel glaucoma; PEX: pseudoexfoliation glaucoma.

### Tissue processing and immunofluorescent staining

The following antibodies were used: rabbit anti-TGF-β-RII (L-21, Santa Cruz Biotechnology, Santa Cruz, CA), and monoclonal mouse anti-PECAM-1 (DAKO, Glostrup, Denmark) to detect vascular endothelial cells; anti-α-SMA, anti-FN, anti-ED-A-FN (Sigma, St.Louis, MO), and anti-O-linked sialoglycoprotein (D2–40, Signet Laboratories, Dedham) to detect lymphatic endothelium; and mouse anti-prolyl-4-hydroxylase (DAKO) directed against an enzyme involved in posttranslational collagen modification to detect activated fibroblasts. Secondary antibodies were labeled with Alexa-488 or Alexa-568 (Molecular Probes, Leiden, The Netherlands). Cryostat sections of 6 µm thickness were prepared, air dried, fixed in pure acetone for 20 min at −20 °C, and blocked for 1 h in TRIS buffered saline (TBS, pH 7.6), containing 4% normal goat serum (NGS, Dianova, Hamburg, Germany). Antibodies were diluted in TBS, containing 1% NGS plus 0.1% Triton X-100. The primary antibodies were incubated overnight at 4 °C in a humidified chamber. Secondary antibodies were applied sequentially for 1 h at room temperature. Following each incubation, the specimens were washed three times in TBS and finally mounted in Vectashield (Vector, Burlingame, CA). The stained slides were viewed with a laser scanning confocal microscope (TCS SP-2, Leica, Bensheim, Germany).

**Figure 1:**
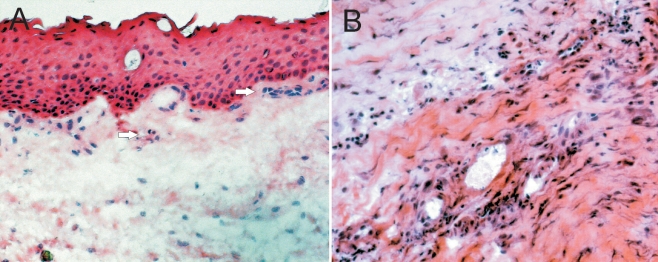
Increased cell density in scarred filtering blebs. Normal conjunctiva (**A**) shows loose subepithelial connective tissue with sparse fibroblasts and occasional vascular structures (arrows). In contrast, scarred filtering blebs (**B**) are characterized by compacted connective tissue with abundant spindle-shaped cells and various vascular structures. H&E stains.

## Results

### Increased cell density in scarred filtering blebs

In normal conjunctiva, the subepithelial stroma contained sparse fibroblasts and occasional small vascular structures (Figure 1A, arrows). Specimens of scarred filtering blebs showed a high density of spindle-shaped cells, presumably fibroblasts, several vascular structures, and areas of inflammatory cell infiltrates (Figure 1B). Only one scar specimen and all normal specimens contained an epithelial layer. To avoid leakage and allow for proper wound closure after revision surgery, care was taken to spare the conjunctival epithelial layer, which explains the absence of epithelium in most scar specimens.

**Figure 2:**
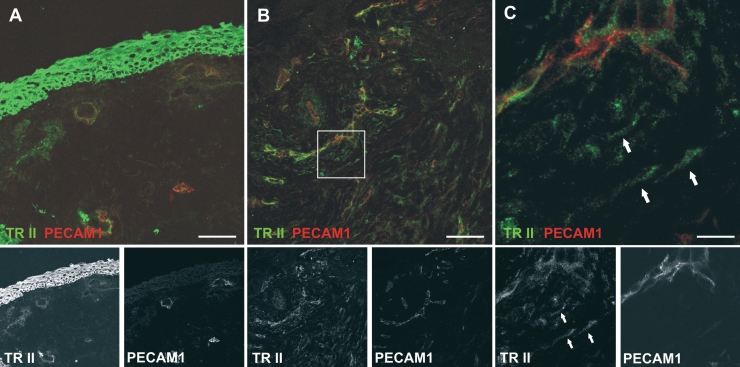
Colocalization of TGF-β-RII and PECAM-1. Native conjunctiva (**A**) and scarred filtering bleb specimens (**B**, **C**) were double-stained for TGF-β-RII (green) and the vascular endothelial cell marker, PECAM-1 (red). **C** is a close-up of a section of **B** as indicated by the frame. Arrows point to elongated TGF-β-RII positive cells devoid of PECAM-1 signal, which are most likely fibroblasts. Scale bar represents 50 µm (**A**, **B**) and 10 µm (**C**).

**Figure 3:**
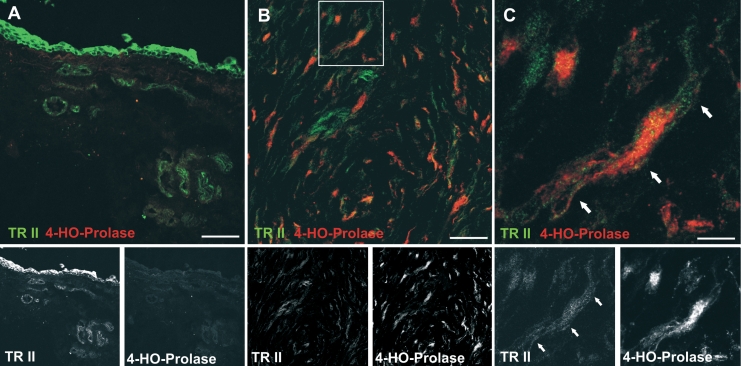
Colocalization of TGF-β-RII and the fibroblast marker prolyl-4-hydroxylase. Native conjunctiva (**A**) and scarred filtering bleb specimens (**B**, **C**) were double-stained for TGF-β-RII (green) and prolyl-4-hydroxylase (red). **C** is a close-up of a portion of **B** as indicated by the frame. Arrows point to a TGF-β-RII positive fibroblast, which are positive for both TGF-β-RII and prolyl-4-hydroxylase. Scale bar represents 50 µm (**A**,**B**) and 10 µm (**C**).

### TGF-β receptor II expression in native conjunctiva and scarred filtering blebs

To assess TGF-β receptor expression and identify TGF-β-responsive cell types, immunofluorescent double stains were performed. Vascular endothelial cells were stained with an antibody recognizing PECAM-1, and activated fibroblasts were detected using an antibody against the enzyme prolyl-4-hydroxylase. TGF-β-RII expression was strong in epithelial cells (Figure 2A) and in vascular endothelial cells (Figure 2A,B,C). Activated, prolyl-4-hydroxylase-positive fibroblasts were absent in normal conjunctiva, and a faint staining for TGF-β-RII was rarely seen in non-vascular areas of the normal conjunctival stroma (Figure 2A and Figure 3A).

**Figure 4:**
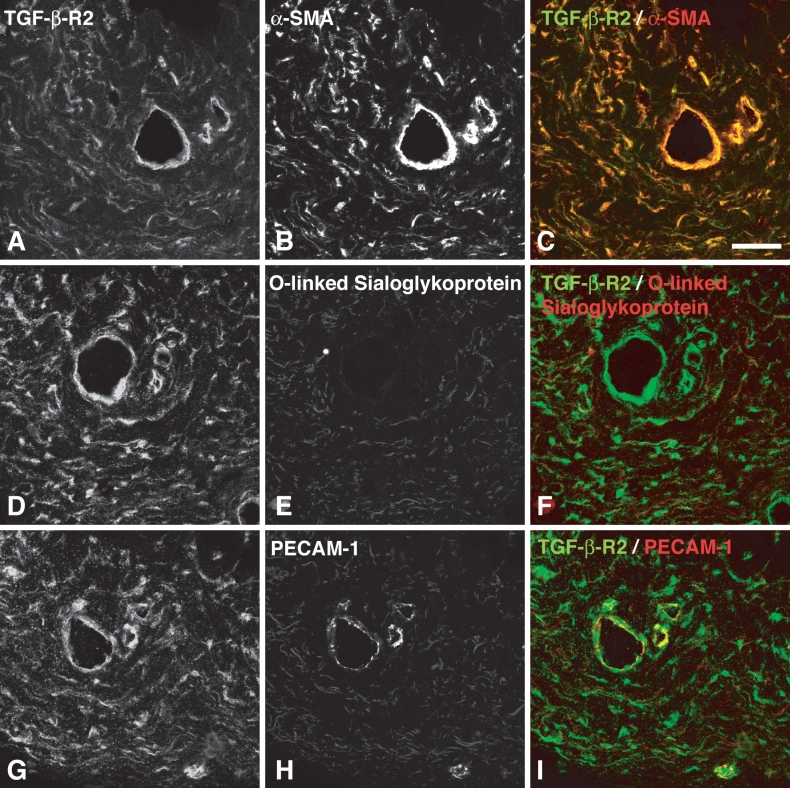
Localization of α-smooth muscle actin (SMA) and the absence of lymphatic endothelium in scarred filtering bleb tissue. Serial sections were double labeled for TGF-β-RII (**A**, **D**, and **G;** green) and α-SMA (**B**, **C**), O-linked sialoglycoprotein (**E**, **F**), and PECAM-1 (**H**, **I**, red). α-SMA is colocalized to TGF-β-RII in stromal fibroblasts and perivascular cells (**A**,**B**,**C**). Vascular structures expressing TGF-β-RII(**H**) were negative for markers of lymphatic endothelium (**E**).Some collagen fiber autofluorescence is present in the red channel (**B**, **E**, **H**), Scale bar represents 50 µm.

Specimens of scarred filtering blebs were rich in vascular structures as detected by PECAM-1 staining (Figure 2B,C). PECAM-1 positive cells stained for TGF-β-RII (Figure 2B,C), but non-vascular spindle-shaped cells were also expressing this receptor (Figure 2C, arrows). The latter appear to be activated fibroblasts as detected by an anti-prolyl-4-hydroxylase antibody (Figure 3B,C). These cells were abundant in scarred filtering blebs, and most of them expressed TGF-β-RII (Figure 3B,C, arrows). In scarred filtering blebs, fibroblasts also expressed α-SMA (Figure 4B,C), which was detected in perivascular cells as well. Lymphatic endothelium was not detected (Figure 4E,F).

No staining was observed when the anti-TGF-β-RII antibody was preadsorbed to a specific control peptide, or when non-immune rabbit IgG was used as a primary antibody as well as when primary antibodies were omitted (data not shown).

### Fibronectin and ED-A fibronectin in native conjunctiva and scarred filtering blebs

**Figure 5:**
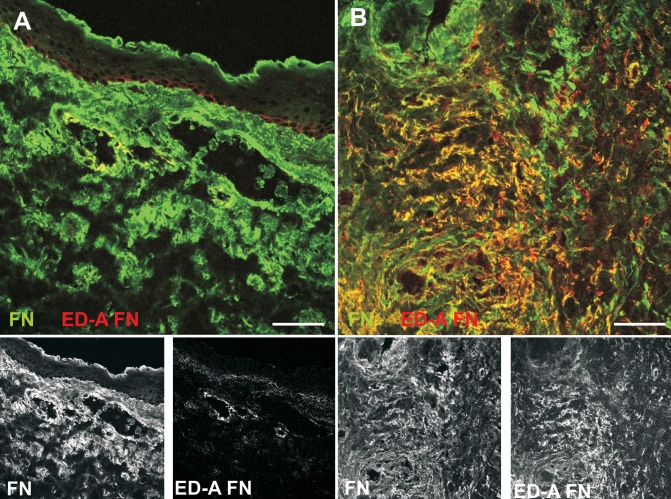
Expression of fibronectin and the ED-A fibronectin splice variant. Fibronectin is abundant in normal conjunctiva (**A**) and in scarred filtering blebs (**B**). In contrast, expression of the fibrogenic ED-A fibronectin splice variant is restricted to perivascular areas in normal conjunctiva (**A**), but expands throughout the tissue in scarred filtering blebs (**B**). Specimens were double-stained for total fibronectin (green) and the fibronectin isoform, ED-A (red). Scale bar represents 50 µm.

To study fibronectin extracellular matrix composition in conjunctival tissue, we performed double stains using antibodies against total fibronectin and the ED-A fibronectin splice variant. Fibronectin was found throughout the subepithelial stroma in both the normal conjunctiva and scarred filtering blebs (Figure 5A,B). In normal conjunctiva, expression of the ED-A fibronectin splice variant was restricted to perivascular areas and basal epithelial cells (Figure 5A). In contrast, ED-A fibronectin was present throughout the stroma of scarred filtering blebs (Figure 5B).

## Discussion

Scar formation remains a serious problem following filtering glaucoma surgery. The cytokine TGF-β, promotes the transdifferentiation of human tenon fibroblasts to myofibroblasts in vitro and has been detected in conjunctival epithelium and subepithelial extracellular matrix after glaucoma surgery [12,13]. However, data on the localization of the respective TGF-β receptors in conjunctival tissue have been lacking. A recent phase III clinical trial, which failed to show an advantage of a topical administered antibody against TGF-β-2 over placebo has raised some reservations regarding the effectiveness of TGF-β-antagonizing strategies to prevent filtering bleb scarring. To further address the relevance of TGF-β in human filtering bleb scarring in vivo, we assessed the cell type-specific localization of TGF-β receptors in normal conjunctiva and scarred filtering blebs. Furthermore, we examined the distribution of ED-A fibronectin as an indicator for TGF-β-induced extracellular matrix alterations.

TGF-β receptors were detected in conjunctival epithelial cells and in vascular endothelial cells of both the normal conjunctiva and scarred filtering blebs. This is consistent with studies of cutaneous squamous epithelium [14] where TGF-β exerts homeostatic and antiproliferative effects [15]. The significance of TGF-β in angiogenesis is also well established although the exact effects of TGF-β on vascular endothelial cells have not fully unraveled. Transgenic mice deficient in TGF-β-1, TGF-β receptor 1 (ALK1), TGF-β receptor 2 (ALK4), or the accessory TGF-β-binding protein endoglin, suffer from defects in angiogenesis and vascular malformations (reviewed in [16,17]). An increase in vascularity is an important clinical sign of impending filtering bleb failure [18].

Most importantly, we provide evidence for a substantially different distribution of TGF-β receptors and ED-A fibronectin in the connective tissue of the normal conjunctiva and scarred filtering blebs. As detected by immunofluorescence, TGF-β receptors were virtually absent in fibroblasts of normal conjunctiva while they were abundant and strongly expressed in activated fibroblasts of scarred filtering blebs. Similarly, ED-A fibronectin was confined to perivascular areas in normal conjunctiva but abundantly present in all the scarred filtering blebs. In light of previous studies by other investigators, these data are consistent with TGF-β-induced alterations and indicate a state of enhanced TGF-β responsiveness in scarred filtering blebs. A strong TGF-β receptor expression can result from stimulation by TGF-β [19,20] and has been demonstrated in subcutaneous fibroblasts of granulation tissue and hypertrophic cutaneous scars [14] as well as in an animal model of excisional wound repair [21]. Furthermore, pronounced TGF-β receptor expression results in enhanced TGF-β responses [22]. The sparse staining for TGF-β-RII in fibroblasts of normal conjunctiva does not exclude TGF-β receptor expression but rather indicates a low expression level close to the limit of immunofluorescence detection. Human tenon fibroblasts, derived from normal conjunctiva, respond to TGF-β in vitro [23], and TGF-β, released during initial wound healing, may well induce increased TGF-β receptor expression and auto-induction of the cytokine TGF-β [24], in conjunctival fibroblasts. Our observation of strong TGF-β receptor expression in subconjunctival fibroblasts of scarred filtering blebs may thus indicate an activated deregulated state of fibroblasts with increased responsiveness to TGF-β. Moreover, the increased expression of prolyl-4-hydroxlase, an enzyme essential in collagen processing, suggests the activation of most fibroblasts in the scarred filtering blebs. The recent observation that four subconjunctival injections of a monoclonal anti-TGF-β2 antibody had no effect on postoperative scarring in a phase III clinical trial [9] does not rule out a role of TGF-β in the scarring process. Our current data and previous studies suggest that the application regimen used in the trial and the concept to distinctly target the TGF-β2 isoform may deserve some reconsideration.

The role of cell-matrix interactions in shaping the response of tissue cells to extracellular stimuli is increasingly acknowledged [25]. Fibronectin is a central component of the extracellular matrix and serves both as a structural and regulatory element. TGF-β induces expression of the fibronectin splice variant, ED-A, which is essential for subsequent TGF-β-induced myofibroblast transdifferentiation [11]. In line with these data, mice deficient for the ED-A fibronectin isoform show impaired wound healing [26]. On the other hand, persistence of activated fibroblasts and ED-A fibronectin is strongly associated with hypertrophic scar formation. It is conceivable that a pronounced TGF-β receptor expression and the presence of ED-A fibronectin enhance TGF-β-driven scarring responses in glaucoma revision surgery.

In summary, our data are consistent with an essential role of TGF-β in human filtering bleb scarring and suggest that specific therapies targeting relevant cell-matrix interactions and TGF-β signaling may be of particular benefit in our care for patients undergoing revision glaucoma surgery.
